# Prevalence of Musculoskeletal Pain in Amateur Golfers in the State of São Paulo: A Cross-sectional Study

**DOI:** 10.1055/s-0044-1786349

**Published:** 2024-06-22

**Authors:** Daniele Rodrigues Gonçalves, Milla Pompilio da Silva, Marcel Jun Sugawara Tamaoki, João Carlos Belloti

**Affiliations:** 1 Departamento de Ortopedia e Traumatologia, Escola Paulista de Medicina, Universidade Federal de São Paulo, São Paulo, SP, Brasil

**Keywords:** cross-sectional studies, golf, musculoskeletal pain, prevalence

## Abstract

**Objective** To verify the prevalence of musculoskeletal pain in amateur golfers in the State of São Paulo, Brazil.

**Methods** The present is a cross-sectional study performed from September 2019 to March 2020 in golf clubs affiliated to Federação Paulista de Golfe (São Paulo Golf Federation). Federation players were evaluated regarding data on golf practice and sport routine by a main investigator, though an assessment form with multiple-choice questions, to determine sample characteristics and recent pain intensity by the Visual Analogue Scale.

**Results** Approximately 359 amateur golfers were analyzed. The prevalence of pain was of 55.15% (95% confidence interval [95%CI]: 50.0% to 60.3%); the average pain intensity according to the VAS was moderate (mean ± standard deviation: 5.21 ± 2.04; odds ration [OR]: 47,98%). The golfer's age range was significantly associated with the presence of pain ( *p* < 0.05). The highest prevalence estimate of pain was of 68.80% in the age group between 30 and 39 years (OR: 7,33; 95%CI: 2,26 to 23,85; *p* = 0,0009). The segments most affected by pain were the upper limbs (65.66%), followed by the spine (59.09%) and the lower limbs (32.83%).

**Conclusion** There is a high prevalence rate of pain in Brazilian amateur golfers, especially in younger players in the age group between 30 and 39 years.

## Introduction

Golf is one of the most popular sports, 1 with 66 million players worldwide. Since 2016 the number of players has increased by over 5.5 million. 2

And like any other sport, it can also lead to musculoskeletal injuries and, consequently, musculoskeletal pain. In a systematic review from 2009, 3 the authors reported that the prevalence of injuries in amateur golfers ranged from 17% to 62%. These injuries occurred throughout the competitive lives of the golfers; however, there may have been a memory bias (when participants did not they accurately remember the events). 4

Additionally, studies on the prevalence of injuries presented other significant biases, such as the failure to mention the sample size calculation, the inclusion and exclusion criteria, and the adopted definition of injury in the sport. 5

In Brazil there are ∼ twenty thousand practitioners of this sport, and recent data on the prevalence of pain is not known in the country. To answer the clinical question about the prevalence of pain in recent golf practice, the performance of an observational study with methodological quality is necessary. 6 7 Therefore, the aim of the present study was to verify prevalence of musculoskeletal pain in amateur golfers in the state of São Paulo.

## Methods

### Study Design

The present was an observational, cross-sectional study conducted in accordance with the Strengthening the Reporting of Observational Studies in Epidemiology (STROBE) statement. 8

### Ethics Committee

The present study involved human participants, and it was approved by the institutional Ethics in Research Committee under CAAE 14666619.4.0000.5505. The participants signed the consent to participate in the study, which was performed according to the Declaration of Helsinki.

### Study Location

The study was conducted in 14 golf clubs located in the state of São Paulo, Brazil, which were affiliated to Federação Paulista de Golfe (São Paulo Golf Federation).

### Eligibility Criteria

The inclusion criteria were Brazilian amateur golfers, affiliated to Federação Paulista de Golfe for more than 1 year, of any sex, and over 18 years of age. Players who underwent medical or physical therapy treatment for orthopedic surgery or bone fracture in the previous year and those who refused to sign the informed consent form were excluded.

### Outcomes

The primary outcome was the prevalence of musculoskeletal pain in the past six months.

### Procedure

An assessment form was developed with thirty questions that, on an average, required five minutes to be filled out. These questions addressed demographic data, data on presence of pain during golf practice in the past six months assessed using a visual analogue scale (VAS), 10 a body diagram of the pain sites, 11 duration of pain, time of pain onset, whether the pain affected swing, time spent away from the game due to the pain, and data about the sport.

Considering that pain is perceived in a specific region of the body, and that it originates in the bones, muscles, ligaments or tendons, and that it can be acute or chronic, 12 the concept of pain used in the present study was pain that occurred during or after golf training or a golf match, regardless of the time off, duration of pain and need for medical attention.

From September 2019 to March 2020, one of the study researchers administered the assessment form in person, and the golfers were approached once after the game. The researcher explained the study, the questionnaire, the selection criteria and the guarantee of data protection and confidentiality to the players. After the application of the eligibility criteria, the golfers who agreed to participate in the research signed the informed consent form.

### Case Set

The participants were selected based on convenience, and the sample size was determined based on the total number of golfers affiliated (4 thousand players) to Federação Paulista de Golfe in 2019. to determine the sample size (representative of this population), the following values were used: 50% of expected pain frequency, 95% confidence interval (95%CI) and 5% sampling error; these measurements yielded a sample size of 350 amateur golfers. 13

### Statistical Analysis

Data were tabulated in a Microsoft Excel 365 spreadsheet (Microsoft Corp., Redmond, WA, United States), and the analyses were performed using the R statistical software (R Foundation for Statistical Computing, Vienna, Austria). Initially, a descriptive analysis of all variables was performed. For the categorical/qualitative variables, absolute and relative frequencies were used, and for the quantitative variables, mean, standard deviation, median, minimum and maximum values and percentages were used. Based on the regression model analysis, the odds ratios (ORs) were estimated with their respective 95%CIs. The Chi-squared and Fisher exact tests were used as categorical variables. For all analyses, the level of significance was set at 5%.

## Results

**Fig. 1:**
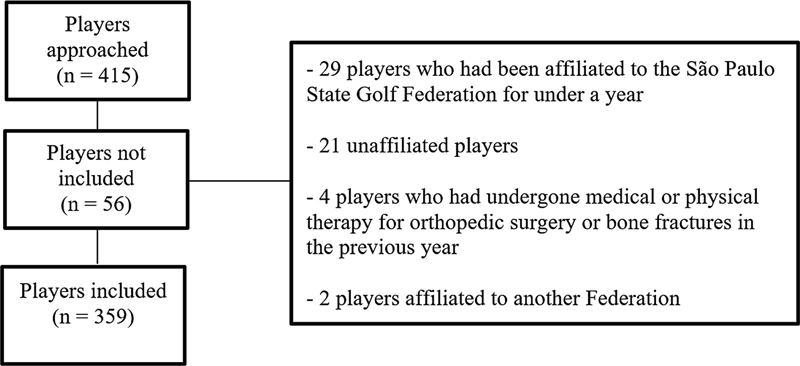
Flowchart for eligibility.

In total, 415 players were approached. However, based on the eligibility criteria, 56 players were excluded from the study, as shown in Fig. 1 .

As a result, 359 amateur golfers were interviewed, and their general characteristics are shown in Table 1 .

**Table 1:** General characteristics of 359 amateur golfers

Numerical variables	Mean(± standard deviation)	Median (minimum value; maximum value)
Age (years)	53.91(± 12.26)	55.00 (21.00; 86.00)
Body mass index (kg/m ^2^ )	26.66(± 03.61)	26.45 (17.97; 50.71)
Years of playing golf	15.04(± 11.77)	12.00 (01.00; 65.00)
Skill level/handicap	18.88(± 07.68)	18.00 (00.00; 40.00)
Tournaments per year	08.06(± 07.77)	6.00 (00.00; 50.00)
Matches per week	02.10(± 00.95)	2.00 (01.00; 06.00)
**Categorical variables**	**Category**	**n (%)**
Sex	Male	311 (86.60%)
	Female	48 (13.40%)
Dominance	Right-handed	338 (94.20%)
	Left-handed	21 (05.80%)
Age group (years)	20–29	11 (03.1%)
	30–39	32 (09.1%)
	40–49	83 (23.5%)
	50–59	113 (32.0%)
	60–69	26 (07.4%)
	≥ 70	
Body mass index (kg/m ^2^ )	18.50–24.99	117 (32.6%)
	25.00–40.00	242 (67.4%)
Weekly visits to the golf course	1–2 times	264 (73.50%)
	3–4 times	87 (24.20%)
	5–6 times	8 (02.20%)
Drive-range training time per week (minutes)	0–30	199 (55.40%)
	31–59	81 (22.60%)
	≥ 60	79 (22.00%)
Putting green practice time per week (minutes)	0–30	269 (74.90%)
	31–59	70 (19.50%)
	≥ 60	20 (05.60%)
Number of drive-range training balls	0–60	241 (67.10%)
	61–120	94 (26.20%)
	≥ 121	24 (06.70%)
Specific physical conditioning for golf	Yes	105 (29.20%)
	No	254 (70.80%)
Other sports	Yes	147 (40,90%)
	No	212 (59,10%)
Warm-up	Yes	220 (61.30%)
	No	139 (38.70%)
Stretching	Yes	68 (18.90%)
	No	291 (81.10%)
Method of carrying the golf bag*	Cart or electric car	126 (35.10%)
	Caddy	117 (32.60%)
	Manually pushing and pulling the cart	111 (30.90%)
	On the shoulder	15 (04.20%)

**Note:** *The total frequency was > 100% because the answer could include more than one category.

As shown in Table 1 , ∼ 87% of the included participants were males and 13% were females, and 94% of the players were right-handed. The mean age of the participants was of 54 ± 12 years, with a higher number of participants in the age group of 50 to 59 years (32%). The analyzed data indicated a mean body mass index (BMI) of 27 ± 4 kg/m ^2^ , with 67.4% of the players being overweight or obese.

The mean number of years of golf experience was of 15 ± 12, and the mean skill level/handicap was of 19 ± 8. Regarding the weekly training time, 75% of the included golfers trained for ≤ 30 minutes on the putting green, 55% trained for 30 minutes on the drive range, and 67% used 0 to 60 balls in the drive-range training.

Overall, 29% of golfers practiced golf-specific physical conditioning, 61% performed warm-up exercises, 19% reported performing stretching exercises, and 40,9% practiced sports other than golf, mainly swimming, tennis, and running.

Table 2 shows that the prevalence of pain in the past 6 months was of 55.15% (95%CI: 50.0% to 60.3%; *n* = 198) among amateur golfers.

**Table 2:** Descriptive analysis of musculoskeletal pain variables during the past 6 months ( *n* = 198)

Numerical variables	Mean ± standard deviation	Median (minimum value; maximum value)
Pain intensity	5.21 ± 2.04	5 (1; 10)
**Categorical variables**	**Category**	**n (%)**
Presence of pain	Yes	198 (55.15%) 95%CI: 50.0–60.3%
Sex	Male	168 (84.80%)
	Female	30 (15.00%)
Pain intensity (Visual Analogue Scale)	1–3.99	56 (28.28%)
	4–6.99	95 (47.98%)
	7–9.99	42 (21.21%)
	10	05 (02.53%)
Pain segment*	Upper limbs	130 (65.66%)
	Spine	117 (59.09%)
	Lower limbs	65 (32.83%)
Site of pain*	Lumbar	96 (48.48%)
	Shoulder	59 (29.80%)
	Elbow	53 (26.77%)
	Knee	26 (13.13%)
	Hip	19 (09.60%)
	Dorsal	15 (07.58%)
	Hand	12 (06.06%)
Time of pain onset*	Playing golf	74 (37.37%)
	After the game	67 (33.83%)
	Unknown	37 (18.68%)
	Coaching golf	25 (12.63%)
	After training	11 (5.56%)
Swing phase*	Acceleration + impact	68 (34.34%)
	Follow-through	60 (30.30%)
	Backswing	42 (21.21%)
	No pain performing these gestures	54 (27.27%)
Duration of pain	< 1 week	73 (36.9%)
	8–30 days	37 (18.7%)
	31–60 days	18 (09.1%)
	61–90 days	29 (14.6%)
	> 90 days	41 (20.7%)
Pain at the time of the interview	No	122 (61.61%)
	Yes	76 (38.39%)

**Abbreviation:** 95%CI, 95% confidence interval.

**Note:** *The total frequency was > 100% because the answer could include more than one category.

The segments most affected by pain in the past 6 months were the upper limbs (65.66%), followed by the spine (59.09%), and the lower limbs (32.83%). However, regarding the site of the pain, the lumbar spine was the most affected (48.48%), followed by the shoulder (29.80%), and the elbow (26.77%). As the players had the option of selecting multiple sites, the total frequency of data exceeded 100%.

When the participants were asked about their pain intensity using the VAS, the intensity category between 4 and 6.99 (moderate pain) was found the most frequent (48%), and the mean pain intensity was found to be of 5.2. The duration of pain was found to be < 1 week for 36.9% of the participants. Of the participants who experienced pain, 50% reported that its onset wsas when they were training for or playing golf.

Table 3 demonstrates the consequences of pain in golfers, with 65,6% not needing to withdraw from training or playing golf because of pain.

**Table 3:** Descriptive analysis of consequences of golf-related musculoskeletal pain in the last 6 months ( *n* = 198)

Variables	Category	n (%)
Healthcare	Yes No	97 (49%) 101 (51%)
Physiotherapy	Yes No	106 (54%) 92 (46%)
Time spent away from training for or playing golf	< 1 week	21 (10.6%)
	8–30 days	31 (15.7%)
	31–90 days	5 (02.5%)
	> 90 days Did not stop	11 (05.6%) 130 (65,6%)
Biomechanical alteration of swing	Yes	106 (53.54%)
	No	92 (46.46%)

As shown in Table 4 , the golfers' age range and specific physical conditioning for golf showed a significant association with the presence of pain ( *p* < 0.05). Golfers aged between 30 and 39 years presented 7.34 (95%CI: 2.24 to 24.06) times more chance of having experienced golf-related pain in the past 6 months than those aged ≥ 70 years ( *p* < 0.05).

**Table 4:** Analysis of factors (related to golf) associated with the presence of pain during the past 6 months among amateur golfers in the state of São Paulo, Brazil ( *n* = 359)

Variable	Category	n (%)	Pain		OR (95%CI)	*p* -value
			Absence: n (%)	Presence: n (%)*		
Age (years)	20–29	11 (3.06%)	6 (54.6%)	05 (45.4%)	2.90 (0.64–13.12)	0.1656
	30–39	32 (8.91%)	10 (31.2%)	22 (68.8%)	7.34 (2.24–24.06)	0.0010**
	40–49	83 (23.1%)	34 (41.0%)	49 (59.0%)	4.86 (1.75–13.45)	0.0024**
	50–59	113 (31.4%)	41 (36.3%)	72 (63.7%)	5.68 (2.10–15.40)	0.0006**
	60–69	88 (24.5%)	45 (51.1%)	43 (48.9%)	3.03 (1.10–08.34)	0.0314**
	> 70	32 (8.91%)	25 (78.1%)	7 (21.9%)	Ref.	
Body mass index (kg/m ^2^ )	18.50–24.99	117 (32.6%)	50 (42.7%)	67 (57.3%)	Ref.	—
	25.00–40.00	242 (67.4%)	111 (45.9%)	131 (54.1%)	0.88 (0.56–1.37)	0.576
Golf experience (years)	≤ 12 ^$^	190 (52.9%)	77 (40.5%)	113 (59.5%)	1.45 (0.96–2.20)	0.0814
	> 12	169 (47.1%)	84 (49.7%)	85 (50.3%)	Ref.	—
Putting green training duration	≤ 30 minutes ^$^	269 (74.9%)	116 (43.1%)	153 (56.9%)	Ref.	
	> 30 minutes	90 (25.1%)	45 (50.0%)	45 (50.0%)	0.76 (0.47–1.22)	0.2568
Drive-range training duration	≤ 30 minutes ^$^	199 (55.4%)	93 (46.7%)	106 (53.3%)	Ref.	
	> 30 minutes	160 (44.6%)	68 (42.5%)	92 (57.5%)	1.19 (0.78–1.80)	0.4229
Physical disability (handicap index)	≤ 18 ^$^	189 (52.6%)	81 (42.9%)	108 (57.1%)	1.18 (0.78–1.80)	0.4243
	> 18	170 (47.4%)	80 (47.1%)	90 (52.9%)	Ref.	
**Method of carrying the golf bag**						
Golf cart	No	233 (64.9%)	102 (43.8%)	131 (56.2%)	1.13 (0.73–1.75)	0.5794
	Yes	126 (35.1%)	59 (46.8%)	67 (53.2%)	Ref.	
Caddy	No	242 (67.4%)	107 (44.2%)	135 (55.8%)	1.08 (0.69–1.68)	0.7289
	Yes	117 (32.6%)	54 (46.2%)	63 (53.8%)	Ref	
Shoulder	No	344 (95.8%)	154 (44.8%)	190 (55.2%)	1.08 (0.38–3.04)	0.8845
	Yes	15 (4.2%)	7 (46.7%)	8 (53.3%)	Ref.	
Manually pulling/pushing the cart	No	248 (69.1%)	116 (46.8%)	132 (53.2%)	0.78 (0.49–1.22)	0.2729
	Yes	111 (30.9%)	45 (40.5%)	66 (59.5%)	Ref.	
Physical conditioning for golf	No	254 (70.8%)	125 (49.2%)	129 (50.8%)	Ref.	
	Yes	105 (29.2%)	36 (34.3%)	69 (65.7%)	1.81 (1.11–2.95)	0.0175**
Warm-up	No	139 (38.7%)	62 (44.6%)	77 (55.4%)	1.02 (0.66–1.56)	0.9415
	Yes	220 (61.3%)	99 (45.0%)	121 (55.0%)	Ref.	
Stretching	No	291 (81.1%)	129 (44.3%)	162 (55.7%)	0.12 (0.66–1.90)	0.6829
	Yes	68 (18.9%)	32 (47.1%)	36 (52.9%)	Ref.	

**Abbreviations:** 95%CI, 95%confidence interval; OR, odds ratio; Ref., reference category of the independent variables.

**Notes:** *Outcome event. ^$^ Median of the sample. **Statistically significant.

Moreover, among golfers who underwent specific physical conditioning for golf, 65.7% experienced pain, whereas among those who did not undergo specific physical conditioning, 50.8% experienced pain. Golfers who underwent specific physical conditioning for golf presented 1.86 (95%CI: 1.16 to 2.98) times more chance of experiencing pain ( *p* < 0.05).

## Discussion

The present is a cross-sectional study which aimed to assess the prevalence of pain in the months before the interview among amateur golfers affiliated to Federação Paulista de Golfe. The period of six months was chosen to reduce memory bias. Hence, the present study was designed employing sample size calculation, eligibility criteria, pain definition, and in-person data collection.

In the present study, 13.4% participants were females and 86.6% were males; these proportions were similar to those found in the literature 14 15 16 17 18 and Federação Paulista de Golfe.

Pain was found to be highly frequent in amateur golfers, with a prevalence of 55.2% in the past 6 months. In the literature, the prevalence of injuries in golfers ranges from 17.1% to 62%. 14 15 16 17 18 19 20 21 These studies analyzed the site of the injury, not the injury itself. Regarding pain intensity, 76.26% of the players were found to have mild-to-moderate pain, with a mean VAS score of 5 to 7. This finding is consistent with an injury study that assessed pain intensity and reported a mean intensity of seven on the VAS. 17

Injury severity can be classified according to how long a player must be away from sport. 22 23 In the present study, 66% of players with pain did not have to stop playing or training golf due to pain, because they reported the pain was mild or not very relevant. This result contradicts that of the study by McHardy et al., 17 in which 55.2% of the injured players had to take breaks of two to three weeks from games or training because of the injury.

On the day of the interview, 38% of the players with pain answered that, although they were in pain, they practiced the sport. This result supports those of the literature that states that injuries can impair golf performance, but they do not necessarily prevent players from playing or competing in the sport. 22 23 The anatomical segments most affected by pain were the upper limbs, followed by the spine and the lower limbs. This result is similar to that of a study by Theriault et al., 15 in which the upper limbs were reported to be the most affected region. However, when analyzing pain in relation to anatomical site, we found that the lumbar spine (48%) was the most commonly affected site, supporting the findings of the aforementioned systematic review 3 and most cross-sectional studies. 17 20 24 25 In the present study, the shoulder was found to be the second site most commonly affected by pain, followed by the elbow. In addition, the right side of golfers was affected by pain in 61% of the cases. Thus, it can be said that the trail side or the right side was more commonly affected, since the sample contained 95% of right-handed participants. Golf is an asymmetrical sport; thus, the muscles on the right and left sides are activated differently. For right-handed golfers, the right side is the trail side and the left side in a right-handed golfer is the lead arm. 26

Some authors state that injuries in amateur golfers may occur because of the biomechanics of the swing movement associated with inadequate techniques, or due to the volume of practice. 26 One of the types of swing is the modern swing, in which greater angular displacement of the lumbar spine occurs, which, in turn, can cause injuries in both professional and amateur golfers. 22 27 In contrast, upper-limb injuries can occur when the club hits a stationary object, such as a rock, a tree root or even hard ground, which results in sudden deceleration of the movement that causes pain and injuries in the region, which may also be related to training volume. 22 27

In golf, the swing is divided into phases: takeaway, backswing, acceleration, impact, early follow-through and late follow-through. 28 In the present study, the phase most associated with pain was the acceleration phase, followed by the follow-through. These findings are inconsistent with those of McHardy et al., 17 who found that 30.2% felt pain during the follow-through phase, and 17% felt pain during the acceleration or impact phases.

The frequency of pain increases with golf play time, training, and playing experience. This may facilitate the onset of pain due to increased exposure/practice volume. Most athletes spent less than 30 minutes per week practicing on the putting green and drive range (54% and 77%, respectively). This may facilitate the onset of pain – not from overuse, but from poor swing technique.

Golfers with a handicap below 18 reported the highest frequency of pain (58.5%); this is similar to the results of studies in the literature, which reported that injuries were more frequent in players with a lower handicap. 13 19 23 The authors of the present study believed that a higher handicap index (less skilled players) was associated with pain. However, in the statistical analysis, no differences were observed between those with higher and lower handicaps (indicating that this parameter was not statistically significant).

Most participants in the present study (67.2%) were overweight or moderately-to-severely obese. This is consistent with a study published in 2020 by Instituto Brasileiro de Geografia e Estatística (IBGE, Brazilian Institute of Geography and Statistics), which reported that 1 in 4 Brazilian adults were obese until 2019. 29 The frequency of pain in the current study was higher in players with a BMI > 25 kg/m ^2^ , with 53.7% of the players being overweight or obese. These data are consistent with thoe of a literature report in which 44.6% of the players were overweight. 21

Approximately 35,1% of golfers preferred using a golf cart over walking around the course; this can lead to a decrease in the beneficial effects of walking. The rise on the use of golf carts may increasingly undermine the inherent health benefits derived from the sport, with negative consequences, such as obesity. 30 In the present study, we believed that the method used to carry the golf bag, especially carrying the bag on a shoulder, and manually pulling and pushing the golf cart, would present a predictive association with pain, but the differences associated with these factors were not statistically significant.

In the present study, age showed a statistically significant association with pain. Golfers who were in the age group of 30 to 69 years presented a greater association with pain, with the category of 30 to 39 years being 7.34 times more likely to experience pain than those aged ≥ 70 years. These results are consistent with those of some studies in the literature, in which golfers over 40 years of age were found to have a greater chance of injury, presenting a risk of injury 5 times greater than those aged > 70 years. 17 18 We believe that the reason why players under 70 years old experience more pain is their desire to hit long distances, using a lot of force in the swing. As a result, they overload the musculoskeletal system and, consequently, cause injuries and pain. Additionally, younger players experience stress at work that can influence the outcome. However, this is a matter that still needs to be investigated.

In the present study, physical conditioning exercises showed a significant association with the frequency of pain. Golfers who perform physical conditioning exercises are 1.86 (95%CI: 1.16–2.98) times more likely to experience pain ( *p* < 0.05). However, details on physical conditioning, duration and frequency per week were not questioned. The players may have begun golf-specific fitness training because of the pain, which could influence the outcome.

With the current prevalence study, we obtained data from amateur golfers that can be used in the development of hypotheses and sample calculation for future prospective studies.

## Limitations of the Study

Since the present is a cross-sectional study, we can report an association, but cannot establish causality, for there is no determination of temporal relationships between exposure and outcome, only the development of hypotheses about the cause or associated factors.

## Conclusion

The prevalence of golf-related pain in the 6 months preceding the assessment was found to be of 55.15%. Pain was more frequent in the upper limb segment, and younger players aged 30 to 69 years were more likely to experience pain than those aged ≥ 70 years.
