# Supplementary material for: Prevalence of Musculoskeletal Pain in Amateur Golfers in the State of São Paulo: A Cross-sectional Study
Source: Rev Bras Ortop (Sao Paulo). 2024 Jun 22;59(3):e420–8. doi: 10.1055/s-0044-1786349 (PMC11193575; doi:10.1055/s-0044-1786349)
Supplement: Related texts

# Research article: Prevalência de dor musculoesquelética em golfistas amadores do estado de São Paulo: Um estudo transversal

**Keywords:** dor musculoesquelética, estudos transversais, golfe, prevalência

## Abstract

**Objetivo** Verificar a prevalência de dores musculoesqueléticas em golfistas amadores do estado de São Paulo.

**Métodos** Este é um estudo transversal realizado de setembro de 2019 a março de 2020 em clubes de golfe afiliados à Federação Paulista de Golfe. Jogadores federados foram avaliados quanto a dados da prática de golfe e da rotina esportiva por um investigador principal, por meio de um formulário de avaliação com questões de múltipla escolha, para a determinação das características da amostra e da intensidade da dor recente pela Escala Visual Analógica (EVA).

**Resultados** Cerca de 359 golfistas amadores foram analisados. A prevalência de dor foi de 55,15% (intervalo de confiança de 95% [IC95%]: 50,0% a 60,3%); a intensidade média da dor segundo a EVA foi moderada (média ± desvio padrão: 5,21 ± 2,04; razão de probabilidades [ *odds ratio* , OR, em inglês]: 47,98%). A faixa etária dos golfistas esteve significativamente associada à presença de dor ( *p* < 0,05). A maior estimativa de prevalência de dor foi de 68,80% na faixa etária de 30 a 39 anos (OR: 7,33; IC95%: 2,26 a 23,85; *p* = 0,0009). Os segmentos mais acometidos por dor foram os membros superiores (65,66%), seguidos da coluna (59,09%) e dos membros inferiores (32,83%).

**Conclusão** Há uma alta taxa de prevalência de dor em golfistas amadores brasileiros, especialmente em jogadores mais jovens, na faixa etária de 30 a 39 anos.

## Introdução

O golfe é um dos esportes mais populares, 1 com 66 milhões de jogadores em todo o mundo. Desde 2016, o número de jogadores aumentou em mais de 5,5 milhões. 2

Como qualquer outro esporte, o golfe também pode causar lesões musculoesqueléticas e, consequentemente, dores musculoesqueléticas. Em uma revisão sistemática de 2009, 3 os autores relataram que a prevalência de lesões em golfistas amadores variou de 17% a 62%. Essas lesões ocorreram ao longo da vida competitiva dos golfistas; no entanto, pode ter havido um viés de memória (quando os participantes não se lembravam com precisão dos eventos). 4

Além disso, estudos sobre prevalência de lesões apresentaram outros vieses significativos, como a falta de menção ao cálculo amostral, aos critérios de inclusão e exclusão e à definição adotada de lesão no esporte. 5

O Brasil tem cerca de vinte mil praticantes de golfe, e dados recentes sobre a prevalência da dor no país não são conhecidos. Para responder à questão clínica sobre a prevalência de dor na prática recente de golfe, é necessário conduzir um estudo observacional com qualidade metodológica. 6 7 Portanto, o objetivo deste estudo foi verificar a prevalência de dor musculoesquelética em golfistas amadores do estado de São Paulo.

## Métodos

## Delineamento Experimental

Este foi um estudo observacional, transversal, realizado de acordo com a declaração *Strengthening the Reporting of Observational Studies in Epidemiology* (STROBE). 8

## Comitê de Ética

Este estudo foi realizado em participantes humanos e foi aprovado pelo Comitê de Ética em Pesquisa institucional sob número CAAE 14666619.4.0000.5505. Os indivíduos assinaram o termo de consentimento livre e esclarecido para a participação no estudo, que foi realizado de acordo com a Declaração de Helsinque.

## Local do Estudo

O estudo foi realizado em 14 clubes de golfe do estado de São Paulo afiliados à Federação Paulista de Golfe.

## Critérios de Elegibilidade

Os critérios de inclusão foram golfistas amadores brasileiros, filiados à Federação Paulista de Golfe havia mais de 1 ano, de qualquer sexo e maiores de 18 anos. Foram excluídos os jogadores que realizaram tratamento médico ou fisioterapêutico por cirurgia ortopédica ou fratura óssea no último ano, e aqueles que se recusaram a assinar o termo de consentimento livre e esclarecido.

## Desfechos

O desfecho primário foi a prevalência de dor musculoesquelética nos últimos seis meses.

## Procedimento

Um formulário de avaliação com trinta questões foi preparado. Responder ao questionário completo levava em média cinco minutos, e as questões abordavam dados demográficos, dados sobre a presença de dor durante a prática de golfe nos últimos 6 meses segundo a escala visual análoga (EVA), 10 um diagrama corporal dos sítios da dor, 11 a duração da dor, o momento de aparecimento da dor, se a dor afetava o *swing* , o tempo afastado do golfe devido à dor e dados sobre o esporte.

Considerando que a dor é percebida em determinada região do corpo, se origina em ossos, músculos, ligamentos ou tendões e pode ser aguda ou crônica, 12 o conceito de dor utilizado neste estudo foi a dor sentida durante ou após um treino ou uma partida de golfe, independentemente do tempo de folga, da duração da dor e da necessidade de atenção médica.

De setembro de 2019 a março de 2020, um dos pesquisadores administrou pessoalmente o formulário de avaliação. Os golfistas foram abordados uma vez após o jogo. O pesquisador explicou o estudo, o questionário, os critérios de seleção e a garantia de proteção e confidencialidade dos dados aos jogadores. Depois de aplicados os critérios de elegibilidade, os golfistas que aceitaram participar da pesquisa assinaram o termo de consentimento livre e esclarecido.

## Série de Casos

Os participantes foram selecionados por conveniência, e o tamanho da amostra foi determinado com base no número total de golfistas filiados (4 mil jogadores) à Federação Paulista de Golfe em 2019. Para a determinação do tamanho da amostra (representativa desta população), foram utilizados os seguintes valores: 50% de frequência esperada de dor, intervalo de confiança de 95% (IC95%) e erro amostral de 5%. Segundo esses valores, o tamanho da amostra foi estabelecido como 350 jogadores de golfe amadores. 13

## Análise Estatística

Os dados foram tabulados em planilha Microsoft Excel 365 (Microsoft Corp., Redmond, WA, Estados Unidos), e as análises foram realizadas no programa estatístico R (R Foundation for Statistical Computing, Viena, Áustria). Inicialmente, foi realizada uma análise descritiva de todas as variáveis. As variáveis categóricas/qualitativas foram expressas como frequências absolutas e relativas, e as variáveis quantitativas, como valores de média, desvio padrão, mediana, mínimos e máximos, e percentuais. Com base na análise do modelo de regressão, foram estimadas as razões de probabilidades ( *odds ratio* , OR, em inglês) com os respectivos IC95%. As variáveis categóricas foram submetidas aos testes do Qui-quadrado e exato de Fisher. O nível de significância de 5% foi considerado em todas as análises.

## Resultados

No total, 415 jogadores foram abordados. Porém, com base nos critérios de elegibilidade, 56 jogadores foram excluídos do estudo, como mostra a Fig. 1 .

**Fig. 1:** Diagrama de elegibilidade.

Assim, foram entrevistados 359 golfistas amadores; suas características gerais são apresentadas na Tabela 1 .

**Tabela 1:** Características gerais dos 359 jogadores amadores de golfe

Variáveis numéricas	Média(± desvio padrão)	Mediana (valor mínimo; valor máximo)
Idade (anos)	53,91(± 12,26)	55,00 (21,00; 86,00)
Índice de massa corporal (kg/m ^2^ )	26,66(± 03,61)	26,45 (17,97; 50,71)
Anos de prática de golfe	15,04(± 11,77)	12,00 (01,00; 65,00)
Nível de habilidade/ *handicap*	18,88(± 07,68)	18,00 (00,00; 40,00)
Torneios por ano	08,06(± 07,77)	6,00 (00,00; 50,00)
Partidas por semana	02,10(± 00,95)	2,00 (01,00; 06,00)
**Variáveis categóricas**	**Categoria**	**n (%)**
Sexo	Masculino	311 (86,60%)
	Feminino	48 (13,40%)
Dominância	Direita	338 (94,20%)
	Esquerda	21 (05,80%)
Faixa etária (anos)	20–29	11 (03,1%)
	30–39	32 (09,1%)
	40–49	83 (23,5%)
	50–59	113 (32,0%)
	60–69	26 (07,4%)
	≥ 70	
Índice de massa corporal (kg/m ^2^ )	18,50–24,99	117 (32,6%)
	25,00–40,00	242 (67,4%)
Visitas semanais ao campo de golfe	1–2 vezes	264 (73,50%)
	3–4 vezes	87 (24,20%)
	5–6 vezes	8 (02,20%)
Tempo de treinamento em *drive range* porsemana (minutos)	0–30	199 (55,40%)
	31–59	81 (22,60%)
	≥ 60	79 (22,00%)
Tempo de treinamento em *putting green* por semana (minutos)	0–30”	269 (74,90%)
	31–59	70 (19,50%)
	≥ 60	20 (05,60%)
Número de bolas no treinamento em *drive range*	0–60 bolas	241 (67,10%)
	61–120 bolas	94 (26,20%)
	≥ 121 bolas	24 (06,70%)
Condicionamento físico específico para o golfe	Sim	105 (29,20%)
	Não	254 (70,80%)
Outros esportes	Sim	147 (40,90%)
	Não	212 (59,10%)
Aquecimento	Sim	220 (61,30%)
	Não	139 (38,70%)
Alongamento	Sim	68 (18,90%)
	Não	291 (81,10%)
Método para carregar a sacola de golfe*	Carrinho ou carro elétrico	126 (35,10%)
	*Caddy*	117 (32,60%)
	Carrinho de tração manual	111 (30,90%)
	Sobre o ombro	15 (04,20%)

**Nota:** *A frequência total foi > 100% porque a resposta podia incluir mais de uma categoria.

Como mostra a Tabela 1 , cerca de 87% dos participantes incluídos eram homens, 13% eram mulheres e 94% dos jogadores eram destros. A média de idade dos participantes foi de 54 ± 12 anos, com maior número de participantes na faixa etária de 50 a 59 anos (32%). Os dados analisados indicaram um índice de massa corporal (IMC) médio de 27 ± 4 kg/m ^2^ , com 67,4% dos jogadores com sobrepeso ou obesidade.

O número médio de anos de experiência no golfe foi de 15 ± 12, e o nível médio de habilidade/ *handicap* foi de 19 ± 8. Em relação ao tempo de treinamento por semana, 75% dos golfistas incluídos treinavam ≤ 30 minutos no *putting green* , 55% treinavam por 30 minutos no *drive range* , e 67% usavam de 0 a 60 bolas no treinamento no *drive range* .

De modo geral, 29% dos golfistas praticavam condicionamento físico específico para o golfe, 61% realizavam exercícios de aquecimento, 19% relataram realizar exercícios de alongamento, e 40,9% praticavam outros esportes além do golfe, em especial natação, tênis e corrida.

A Tabela 2 mostra que a prevalência de dor nos últimos 6 meses foi de 55,15% (IC95%: 50,0% a 60,3%; n = 198) entre os golfistas amadores.

**Tabela 2:** Análise descritiva das variáveis de dor musculoesquelética nos últimos 6 meses ( *n* = 198)

Variáveis numéricas	Média ± desvio-padrão	Mediana (valor mínimo; valor máximo)
Intensidade da dor	5,21 ± 2,04	5 (1; 10)
**Variáveis categóricas**	**Categoria**	**n (%)**
Presença de dor	Sim	198 (55,15%) IC95%: 50,0–60,3%
Sexo	Masculino	168 (84,80%)
	Feminino	30 (15,00%)
Intensidade de dor (Escala Visual analógica)	1–3,99	56 (28,28%)
	4–6,99	95 (47,98%)
	7–9,99	42 (21,21%)
	10	05 (02,53%)
Segmento com dor*	Membros superiores	130 (65,66%)
	Coluna	117 (59,09%)
	Membros inferiores	65 (32,83%)
Sítio de dor*	Lombar	96 (48,48%)
	Ombro	59 (29,80%)
	Cotovelo	53 (26,77%)
	Joelho	26 (13,13%)
	Quadril	19 (09,60%)
	Dorsal	15 (07,58%)
	Mão	12 (06,06%)
Momento de aparecimento da dor*	Jogando golfe	74 (37,37%)
	Após o jogo	67 (33,83%)
	Desconhecido	37 (18,68%)
	Treinando golfe	25 (12,63%)
	Após o treinamento	11 (5,56%)
Fase do *swing* *	Aceleração + impacto	68 (34,34%)
	*Follow-through*	60 (30,30%)
	*Backswing*	42 (21,21%)
	Sem dor durante estes gestos	54 (27,27%)
Duração da dor	< 1 semana	73 (36,9%)
	8–30 dias	37 (18,7%)
	31–60 dias	18 (09,1%)
	61–90 dias	29 (14,6%)
	> 90 dias	41 (20,7%)
Dor no momento da entrevista	Não	122 (61,61%)
	Sim	76 (38,39%)

**Abreviatura:** IC95%, intervalo de confiança de 95%.

**Nota:** *A frequência total foi > 100% porque a resposta podia incluir mais de uma categoria.

Os segmentos mais acometidos por dor nos últimos 6 meses foram os membros superiores (65,66%), seguidos da coluna (59,09%) e dos membros inferiores (32,83%). Porém, quanto ao sítio da dor, o mais acometido foi a coluna lombar (48,48%), seguida do ombro (29,80%) e do cotovelo (26,77%). Como os jogadores tinham a opção de selecionar vários segmentos, a frequência total dos dados foi superior a 100%.

Quando os participantes foram questionados sobre a intensidade da dor por meio da EVA, a categoria de intensidade entre 4 e 6,99 pontos (dor moderada) foi considerada a mais frequente (48%); a intensidade média da dor foi de 5,2 pontos. A duração da dor foi inferior a 1 semana em 36,9% dos participantes. Dos participantes que sentiram dor, 50% relataram seu aparecimento durante o treino ou jogo de golfe.

A Tabela 3 mostra as consequências da dor em golfistas; 65,6% dos entrevistados não precisaram interromper o treino ou o jogo de golfe por causa da dor.

**Tabela 3:** Análise descritiva das consequências da dor musculoesquelética relacionada ao golfe nos últimos 6 meses ( *n* = 198)

Variáveis	Categoria	n (%)
Cuidados de saúde	Sim Não	97 (49%) 101 (51%)
Fisioterapia	Sim Não	106 (54%) 92 (46%)
Tempo sem treinar ou jogar golfe	< 1 semana	21 (10,6%)
	8–30 dias	31 (15,7%)
	31–90 dias	5 (02,5%)
	> 90 dias Sem interrupção	11 (05,6%) 130 (65,6%)
Alteração biomecânica do *swing*	Sim	106 (53,54%)
	Não	92 (46,46%)

Como mostra a Tabela 4 , a faixa etária dos golfistas e o condicionamento físico específico para o golfe apresentaram associação significativa com a presença de dor ( *p* < 0,05). Golfistas entre 30 e 39 anos de idade tiveram 7,34 (IC95%: 2,24 a 24,06) vezes mais chances de sentir dor relacionada ao golfe nos últimos 6 meses do que aqueles com idade ≥ 70 anos ( *p* < 0,05).

**Tabela 4:** Análise dos fatores (relacionados ao golfe) associados à presença de dor nos últimos 6 meses entre golfistas amadores do estado de São Paulo, Brasil ( *n* = 359)

Variável	Categoria	n (%)	Dor		OR (IC95%)	Valor de *p*
			Ausente: n (%)	Presente: n (%)*		
Idade (anos)	20–29	11 (3,06%)	6 (54,6%)	05 (45,4%)	2,90 (0,64–13,12)	0,1656
	30–39	32 (8,91%)	10 (31,2%)	22 (68,8%)	7,34 (2,24–24,06)	0,0010**
	40–49	83 (23,1%)	34 (41,0%)	49 (59,0%)	4,86 (1,75–13,45)	0,0024**
	50–59	113 (31,4%)	41 (36,3%)	72 (63,7%)	5,68 (2,10–15,40)	0,0006**
	60–69	88 (24,5%)	45 (51,1%)	43 (48,9%)	3,03 (1,10–08,34)	0,0314**
	> 70	32 (8,91%)	25 (78,1%)	7 (21,9%)	Ref.	
Índice de massa corporal (kg/m ^2^ )	18.50–24.99	117 (32,6%)	50 (42,7%)	67 (57,3%)	Ref.	—
	25.00–40.00	242 (67,4%)	111 (45,9%)	131 (54,1%)	0,88 (0,56–1,37)	0,576
Experiência de golfe (anos)	≤ 12 ^$^	190 (52,9%)	77 (40,5%)	113 (59,5%)	1,45 (0,96–2,20)	0,0814
	> 12	169 (47,1%)	84 (49,7%)	85 (50,3%)	Ref.	—
Duração do treinamento em *putting green*	≤ 30 minutos ^$^	269 (74,9%)	116 (43,1%)	153 (56,9%)	Ref.	
	> 30 minutos	90 (25,1%)	45 (50,0%)	45 (50,0%)	0,76 (0,47–1,22)	0,2568
Duração do treinamento em *drive range*	≤ 30 minutos ^$^	199 (55,4%)	93 (46,7%)	106 (53,3%)	Ref.	
	> 30 minutos	160 (44,6%)	68 (42,5%)	92 (57,5%)	1,19 (0,78–1,80)	0,4229
Incapacidade física (índice de *handicap* )	≤ 18 ^$^	189 (52,6%)	81 (42,9%)	108 (57,1%)	1,18 (0,78–1,80)	0,4243
	> 18	170 (47,4%)	80 (47,1%)	90 (52,9%)	Ref.	
**Método para carregar a sacola de golfe**						
Carrinho de golfe	Não	233 (64,9%)	102 (43,8%)	131 (56,2%)	1,13 (0,73–1,75)	0,5794
	Sim	126 (35,1%)	59 (46,8%)	67 (53,2%)	Ref	
*Caddy*	Não	242 (67,4%)	107 (44,2%)	135 (55,8%)	1,08 (0,69–1,68)	0,7289
	Sim	117 (32,6%)	54 (46,2%)	63 (53,8%)	Ref	
Ombro	Não	344 (95,8%)	154 (44,8%)	190 (55,2%)	1,08 (0,38–3,04)	0,8845
	Sim	15 (4,2%)	7 (46,7%)	8 (53,3%)	Ref.	
Carrinho de tração manual	Não	248 (69,1%)	116 (46,8%)	132 (53,2%)	0,78 (0,49–1,22)	0,2729
	Sim	111 (30,9%)	45 (40,5%)	66 (59,5%)	Ref.	
Condicionamento físico para o golfe	Não	254 (70,8%)	125 (49,2%)	129 (50,8%)	Ref.	
	Sim	105 (29,2%)	36 (34,3%)	69 (65,7%)	1,81 (1,11–2,95)	0,0175**
Aquecimento	Não	139 (38,7%)	62 (44,6%)	77 (55,4%)	1,02 (0,66–1,56)	0,9415
	Sim	220 (61,3%)	99 (45,0%)	121 (55,0%)	Ref.	
Alongamento	Não	291 (81,1%)	129 (44,3%)	162 (55,7%)	0,12 (0,66–1,90)	0,6829
	Sim	68 (18,9%)	32 (47,1%)	36 (52,9%)	Ref.	

**Abreviaturas:** IC95%, intervalo de confiança de 95%; OR, *odds ratio* (razão de probabilidades); Ref., categoria de referência das variáveis independentes.

**Notas:** *Evento de desfecho. ^$^ Mediana da amostra. **Estatisticamente significativo.

Além disso, entre os golfistas que realizavam condicionamento físico específico para golfe, 65,7% sentiram dor, ao passo que, entre os que não realizaram condicionamento físico específico, 50,8% sentiram dor. Golfistas que realizavam condicionamento físico específico para golfe tiveram 1,86 (IC95%: 1,16 a 2,98) vezes mais chance de sentir dor ( *p* < 0,05).

## Discussão

Este é um estudo transversal com objetivo de avaliar a prevalência de dor nos seis meses anteriores ao momento da entrevista em golfistas amadores filiados à Federação Paulista de Golfe. O período de seis meses foi escolhido para reduzir o viés de memória. Portanto, este estudo foi delineado empregando cálculo de tamanho amostral, critérios de elegibilidade, definição de dor e coleta presencial de dados.

Neste estudo, 13,4% dos participantes eram do sexo feminino e 86,6%, do sexo masculino, proporções semelhantes às encontradas na literatura 14 15 16 17 18 e na Federação Paulista de Golfe.

A dor foi muito frequente em golfistas amadores, com prevalência de 55,2% nos últimos 6 meses. Na literatura, a prevalência de lesões em golfistas varia de 17,1% a 62%. 14 15 16 17 18 19 20 21 Esses estudos analisaram a localização da lesão, não a lesão em si. Em relação à intensidade da dor, 76,26% dos jogadores apresentaram dor leve a moderada, com pontuação média na EVA entre 5 e 7. Este achado condiz com um estudo que avaliou a intensidade da dor e indicou uma intensidade média de 7 pontos na EVA. 17

A gravidade da lesão pode ser classificada de acordo com o tempo que um jogador deve ficar afastado do esporte. 22 23 No presente estudo, 66% dos jogadores com dor não tiveram que parar de jogar ou de treinar golfe por causa da dor, pois relataram que a dor era branda ou não muito relevante. Este resultado contradiz o estudo de McHardy et al., 17 em que 55,2% dos jogadores lesionados tiveram que fazer uma pausa de 2 a 3 semanas nos jogos ou treinos por causa da lesão.

No dia da entrevista, 38% dos jogadores com dor responderam que praticavam o esporte embora sentissem dor. Esse resultado corrobora a literatura que afirma que as lesões podem prejudicar o desempenho no golfe, mas não necessariamente impedem os atletas de jogar ou competir no esporte. 22 23 Os segmentos anatômicos mais acometidos pela dor foram os membros superiores, seguidos da coluna vertebral e dos membros inferiores. Esse resultado é semelhante ao do estudo de Theriault et al., 15 em que os membros superiores foram a região mais afetada. Porém, ao analisar a dor em relação ao sítio anatômico, descobriu-se que a coluna lombar (48%) foi o local mais acometido, o que corrobora os achados da supracitada revisão sistemática 3 e da maioria dos estudos transversais. 17 20 24 25 No presente estudo, o ombro foi o segundo sítio mais acometido pela dor, seguido pelo cotovelo. Além disso, o lado direito dos golfistas foi afetado pela dor em 61% dos casos. Assim, pode-se afirmar que o lado da trilha ou o lado direito foi mais acometido, uma vez que a amostra continha 95% de participantes destros. O golfe é um esporte assimétrico; assim, os músculos dos lados direito e esquerdo são ativados de forma diferente. Para golfistas destros, o lado direito é o lado da trilha e o lado esquerdo em um golfista destro é o braço principal. 26

Alguns autores afirmam que lesões em golfistas amadores podem ocorrer devido à biomecânica do movimento do *swing* associada a técnicas inadequadas, ou devido ao volume de prática. 26 Um dos tipos de *swing* é o *swing* moderno, em que há maior deslocamento angular da coluna lombar, o que, por sua vez, pode causar lesões em golfistas profissionais e amadores. 22 27 Por outro lado, lesões nos membros superiores podem ocorrer quando o taco atinge um objeto estacionário, como uma pedra, uma raiz de árvore ou até mesmo solo duro, o que causa desaceleração repentina do movimento e gera dores e lesões na região, que também podem estar relacionadas ao volume de treino. 22 27

No golfe, o *swing* é dividido em fases: *takeaway* , *backswing* , aceleração, impacto, *follow-through* inicial e *follow-through* tardio. 28 No presente estudo, a fase mais associada à dor foi a de aceleração seguida, seguida da de *follow-through* . Esses achados são inconsistentes com os de McHardy et al., 17 que observaram que 30,2% dos atletas sentiram dor durante a fase de *follow-through* e 17% sentiram dor durante a fases de aceleração ou de impacto.

A frequência da dor aumenta com o tempo de jogo de golfe, o treinamento e a experiência no esporte. Isso pode facilitar o aparecimento da dor devido ao aumento do volume de exposição/prática. A maioria dos atletas passou menos de 30 minutos por semana praticando no *putting green* e no *drive range* (54% e 77%, respectivamente). Isto talvez possa facilitar o aparecimento da dor, não devido ao uso excessivo, mas em decorrência da má técnica de *swing* .

Os golfistas com *handicap* inferior a 18 relataram maior frequência de dor (58,5%); isso é semelhante aos resultados de estudos da literatura, que relataram que as lesões eram mais frequentes em jogadores com menor *handicap* . 13 19 23 Acreditávamos que um maior índice de *handicap* (jogadores menos habilidosos) estava associado à dor. No entanto, na análise estatística, não foram detectadas diferenças entre aqueles com *handicaps* superiores e inferiores, o que indica que este parâmetro não foi estatisticamente significativo.

A maioria dos participantes do estudo (67,2%) estava com sobrepeso ou apresentava obesidade de moderada a grave. Isso condiz com um estudo publicado em 2020 pelo Instituto Brasileiro de Geografia e Estatística (IBGE), 29 que relatou que 1 em cada 4 adultos brasileiros era obeso até 2019. A frequência de dor no presente estudo foi maior em jogadores com IMC > 25 kg/m ^2^ , e 53,7% dos jogadores tinham sobrepeso ou obesidade. Esses dados são consistentes com os de artigos da literatura, em que 44,6% dos jogadores estavam acima do peso. 21

Aproximadamente 35,1% dos jogadores de golfe preferiam usar um carrinho de golfe a caminhar pelo campo; isso pode diminuir os efeitos benéficos da caminhada. O uso crescente de carrinhos de golfe pode minar cada vez mais os benefícios inerentes à saúde decorrentes do esporte, com consequências negativas, como a obesidade. 30 Neste estudo, acreditou-se que o método utilizado para transportar a sacola de golfe, principalmente em um ombro, e puxar e empurrar manualmente o carro teriam uma associação preditiva com a dor, mas as diferenças associadas a estes fatores não foram estatisticamente significativas.

Neste estudo, a idade apresentou associação estatisticamente significativa com a dor. Golfistas na faixa etária de 30 a 69 anos tiveram maior associação com dor, sendo que a categoria de 30 a 39 anos teve 7,34 vezes mais chances de sentir dor do que aqueles com idade ≥ 70 anos. Esses resultados condizem com alguns estudos da literatura que observaram que golfistas com mais de 40 anos de idade tinham maior chance de lesão, com risco 5 vezes maior de lesão do que aqueles com idade > 70 anos. 17 18 Acreditamos que o motivo pelo qual jogadores com menos de 70 anos sentem mais dor é a vontade de atingir longas distâncias, oi que os leva a usar muita força no *swing* . Com isso, sobrecarregam o sistema musculoesquelético e, consequentemente, causam lesões e dores. Além disso, os jogadores mais jovens vivenciam estresse no trabalho, o que pode influenciar o desfecho. Contudo, este é um assunto que ainda precisa ser investigado.

No presente estudo, a preparação física apresentou associação significativa com a frequência de dor. Golfistas que fazem preparação física têm 1,86 (IC95%: 1,16 a 2,98) vezes mais chance de sentir dor ( *p* < 0,05). Porém, detalhes sobre condicionamento físico, duração e frequência semanal não foram questionados. Os jogadores talvez tenham começado a fazer exercícios específicos para o golfe por causa da dor, o que pode influenciar o desfecho.

Este estudo de prevalência obteve dados de golfistas amadores que poderão ser utilizados na elaboração de hipóteses e cálculo amostral para futuros estudos prospectivos.

## Limitações do Estudo

Por se tratar de um estudo transversal, pode-se relatar uma associação, mas não estabelecer causalidade, pois não há determinação de relações temporais entre a exposição e o desfecho, apenas geração de hipóteses sobre a causa ou fatores associados.

## Conclusão

A prevalência de dor relacionada ao golfe nos 6 meses anteriores à avaliação foi de 55,15%. A dor foi mais frequente nos membros superiores, e os jogadores mais jovens, com idade entre 30 e 69 anos, tiveram maior chance de sentir dor do que aqueles com idade ≥ 70 anos.
